# Peripheral inflammatory factors as prognostic predictors for first-line PD-1/PD-L1 inhibitors in advanced non-small cell lung cancer

**DOI:** 10.1038/s41598-024-84469-y

**Published:** 2025-04-02

**Authors:** Chen-xing Jin, Yan-song Liu, He-nan Qin, Yi-bin Teng, Rui Sun, Zhong-jing Ma, A-man Wang, Ji-wei Liu

**Affiliations:** 1 Department of Oncology, The First Affiliated Hospital of Dalian Medical University, Dalian, 116011 Liaoning China; 2 Department of Anesthesiology, The First Affiliated Hospital of Dalian Medical University, Dalian, 116011 Liaoning China; 3 Present Address: Department of Anesthesiology, Obstetrics & Gynecology Hospital of Fudan University, Shanghai, 200011 Shanghai China

**Keywords:** Non-small cell lung cancer, Immune checkpoint inhibitors, Biomarkers, Immunotherapy respond, Neutrophil to lymphocyte ratio, Systemic inflammatory index, Predictive markers, Prognostic markers

## Abstract

Immune checkpoint inhibitors (ICIs) have significantly improved the efficacy and prognosis of patients with non-small cell lung cancer (NSCLC). However, there remains a lack of optimal predictive biomarkers for assessing the response of ICIs. This study aimed to evaluate peripheral inflammatory factors as potential predictive biomarkers for NSCLC patients treated with ICIs. We retrospectively analyzed the correlation between peripheral inflammatory factors and the efficacy and prognosis of 124 patients with driver gene-negative advanced NSCLC who received first-line ICIs at our center from September 2018 to June 2022. Progression-free survival (PFS) was estimated using the Kaplan–Meier method. The association between the factors and multiple endpoints were investigated using univariate and multivariate analyses. A total of 124 patients were enrolled in this study. The objective response rate (ORR) was 49.2% and the disease control rate (DCR) was 97.6%, respectively. The median PFS was 12.7 months. The ORR differed statistically between groups based on the NLR, SII, with higher ORR observed in patients with an NLR ratio < 0.68, SII at 6 weeks < 531.26, and SII ratio < 0.74 (*p* < 0.05). The univariate analysis indicated that ECOG 0–1, smoking, NLR at 6 weeks < 2.72, NLR ratio < 0.68, LMR < 1.34, LMR ratio $\ge$ 1.38, and SII at 6 weeks < 531.26 were associated with longer PFS (*p* < 0.05). The multivariate analysis revealed that smoking (*p* = 0.013), baseline LMR (*p* = 0.015), and SII at 6 weeks (*p* = 0.010) were independent predictors of PFS. NLR, LMR, and SII maybe biomarkers for predicting the efficacy and prognosis of first-line ICIs therapy in driver gene-negative advanced NSCLC.

## Introduction

In recent years, immune checkpoint inhibitors (ICIs) that target programmed death 1 (PD-1) or its ligand (PD-L1) has significantly improved the prognosis of advanced non-small cell lung cancer (NSCLC) compared with standard chemotherapy. The KEYNOTE series of studies demonstrated that first-line therapy with pembrolizumab prolonged overall survival (OS), with a median OS of 30 months and a 5-year OS rate of 31.9% in patients with high PD-L1 expression^1–3^. The IMpower110 study showed that for patients with high PD-L1 expression, first-line therapy with atezolizumab significantly prolonged both progression-free survival (PFS) and OS^4^. In phase III clinical trials of first-line ICIs combined with chemotherapy, patients experienced significant benefits in objective response rate (ORR), PFS, and OS, regardless of PD-L1 expression, with a 3-year OS rate of approximately 30%^5^. However, despite the promising results of ICIs in advanced NSCLC, only a limited proportion of patients achieve long-term survival benefits. Furthermore, immune-related adverse events (IRAEs), which are specific to immunotherapy, pose potential risks of morbidity and mortality^6^.

The expression of PD-L1 in the tumor tissue is the most widely used biomarker^7^. The KEYNOTE-024 and KEYNOTE-042 studies demonstrated superior clinical benefits of pembrolizumab monotherapy versus platinum-based chemotherapy in unresectable locally advanced or metastatic NSCLC patients with a PD-L1 tumor proportion score (TPS) of ≥ 1%^8,9^. Notably, patients with a PD-L1 TPS of ≥ 50% showed significant improvements in both PFS and OS. Furthermore, the KEYNOTE-189 and KEYNOTE-407 studies indicated that pembrolizumab combined with platinum-based doublet chemotherapy significantly prolonged PFS and OS compared with chemotherapy alone in metastatic NSCLC patients^10,11^. The clinical benefit of immunotherapy increased with higher thresholds of tumor-membrane expression. However, the lack of an association between the benefit and PD-L1 expression levels in squamous NSCLC patients was confusing^12^. The overall survival benefit was present in the PD-L1 low-expressing or absent groups, which pointed out the imprecision of the PD-L1 as a biomarker^13^. When a combination of chemotherapy and immunotherapy was used in the first line, the effect of PD-L1 expression as a biomarker was impaired^14,15^. Due to heterogeneity in the association between PD-L1 expression on tumor cells and the clinical response to ICIs, it is essential to establish biomarkers that help predict which patients will benefit from these therapies. Tumor mutational burden (TMB), microsatellite instability-high (MSI-H), deficient mismatch repair (dMMR), tumor infiltrating lymphocytes (TIL), intestinal microbiota has also been suggested as potential biomarkers for the response to ICIs^16,17^. However, these biomarkers have several limitations, and none have been confirmed as reliable predictors of response in these patients.

Several studies have suggested that tumor-associated inflammation plays a decisive role in the immune response of patients with malignant neoplasms and could be applied to immunotherapy^18,19^. In this content, the research first started with nonspecific biomarkers like neutrophil to lymphocyte ratio (NLR) reflecting the general status of the immune system and expanded to different blood-derived parameters related to the systemic inflammatory response^20^, such as lymphocyte to monocyte ratio (LMR)^21^, systemic immune-inflammation index (SII)^22^. However, their clinical application as biomarkers for ICIs remains to be further validated.

To explore convenient and feasible biomarkers for ICIs as first-line therapy in NSCLC, the main objective of our study was to analyze the relationship between peripheral inflammatory factors, including NLR, LMR, and SII, and the efficacy and prognosis of driver gene-negative advanced NSCLC patients, which may provide theoretical basis for screening the population benefiting from ICIs treatment.

## Materials and methods

### Patient selection

**Fig. 1:**
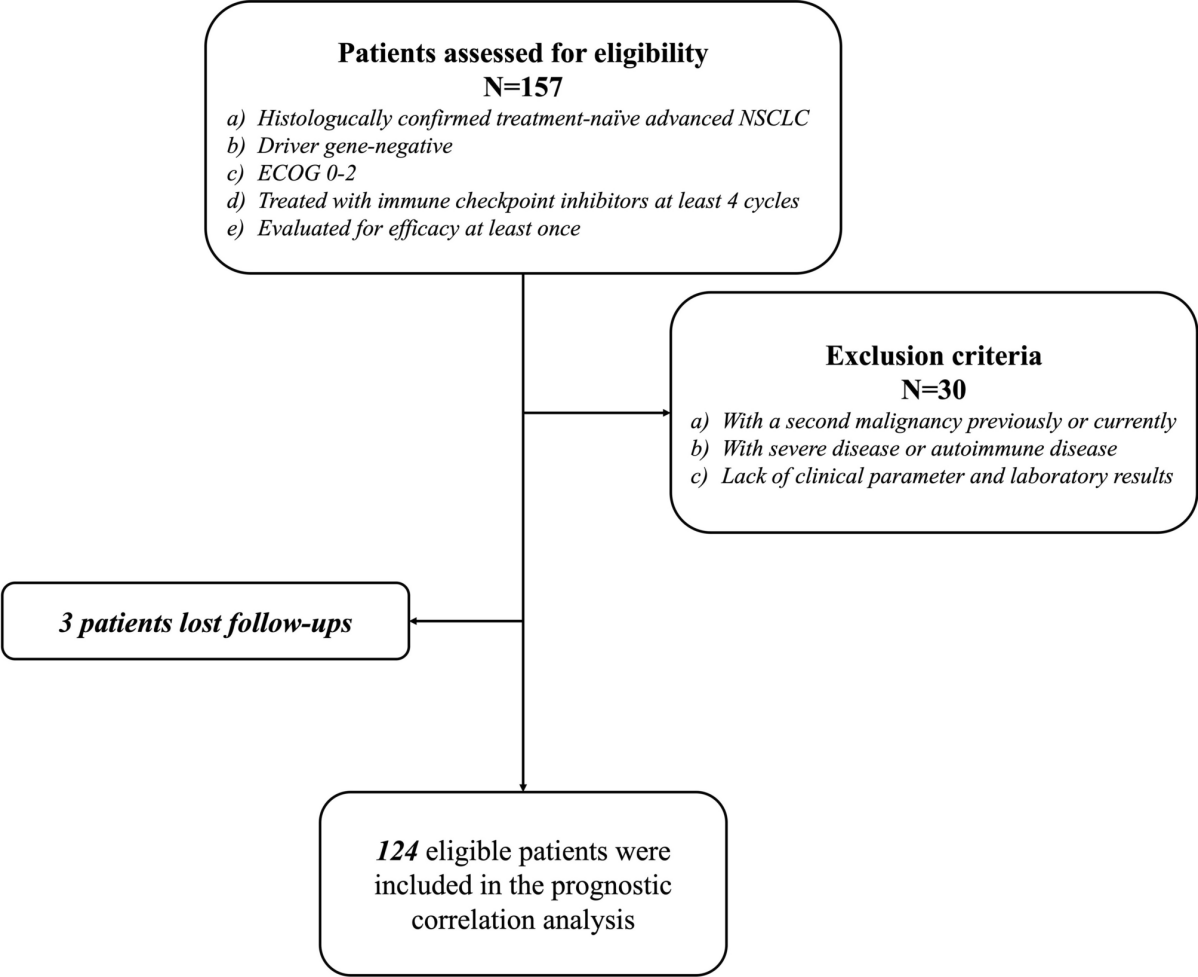
The flowchart of this study. NSCLC, Non-small Cell Lung Cancer; ECOG, Eastern Cooperative Oncology Group Performance Status.

The study included patients with advanced NSCLC who received ICIs as a first-line treatment in The First Affiliated Hospital of Dalian Medical University from September 2018 to June 2022. The eligible patients must be histologically confirmed treatment-naïve driver gene-negative stage IIIB/C and IV NSCLC, with at least one measurable target lesion for response evaluation, an Eastern Cooperative Oncology Group Performance Status score of 0–2. Patients received at least 4 cycles of ICIs and were evaluated for efficacy at least once. We excluded patients with a second primary malignancy, severe disease, and autoimmune disease. A flowchart for the study is presented in Fig. 1. This study was conducted in accordance with the Declaration of Helsinki and was approved by the Ethics Committee of the First Affiliated Hospital of Dalian Medical University (IRB No. PJ-KS-KY-2023-312).

### ICIs treatment

All the patients enrolled in this study received intravenous immune checkpoint inhibitors, including camrelizumab, nivolumab, pembrolizumab, toripalimab, sintilimab, and atezolizumab. Either ICIs monotherapy or in combination with chemotherapy can be enrolled. Every 21 to 28 days represented a treatment cycle. Routine blood tests were performed within 7 days before each cycle of immunotherapy. Treatment was continued until disease progression, death, and treatment intolerance-related adverse effects.

### Response evaluation

The last follow-up date was December 31, 2023. Tumor response was evaluated every 4–6 weeks by modified Response Evaluation Criteria in Solid Tumors (mRECIST) and was assessed as complete response (CR), partial response (PR), stable disease (SD), or progressive disease (PD). ORR was defined as the ratio of the sum of CR plus PR. Disease control rate (DCR) was defined as the ratio of the sum of CR and PR and SD. Progression-free survival (PFS) was defined as the time from the first treatment cycle with ICIs to radiographically recorded disease progression or death or the last follow-up. Duration of response (DoR) was defined as the time from the first documented best overall response CR or PR to date of radiologically confirmed disease progression or death from any cause.

### Follow up

To assess survival outcomes, scheduled outpatient visits or telephone follow-up were performed every 3 months. Data obtained from electronic medical records and pharmacy databases included patient demographic information and clinical data, hematologic and biochemical parameters at baseline (before the first cycle), concomitant treatments, treatment response, and the progression information at the last follow-up date.

### Peripheral inflammatory factors

NLR was defined as absolute neutrophil count/total lymphocyte count; LMR was defined as total lymphocyte count/monocyte count; SII was defined as platelet × neutrophil count/total lymphocyte count; NLR ratio was defined as NLR at 6 weeks/baseline NLR; LMR ratio was defined as LMR at 6 weeks/baseline LMR; SII ratio was defined as SII at 6 weeks/baseline SII. The cutoff value for baseline NLR, LMR, and SII were 3.61, 1.34, 1722.60; NLR at 6 weeks, LMR at 6 weeks, and SII at 6 weeks were 2.72, 2.58, 531.26; NLR ratio, LMR ratio, and SII ratio were 0.68, 1.38, 0.74, respectively, which were determined using the ROC curve.

### Statistical analysis

The chi-squared test and Fisher’s exact test were used to assess the relationship among categorical variables. The Kaplan–Meier method was used to assess the PFS. For the evaluation of independent prognostic factors, both univariate analysis and multivariate analysis were performed. The results are expressed as hazard ratio (HR) 95% CI, and *p* values < 0.05 were considered statistically significant. Candidate variables with a *p* value < 0.2 on univariate analysis were entered into a multivariable Cox proportional-hazards model. Statistical analyses were performed using SPSS v26.0 (IBM, Armonk, NY, United States) and the figure for this study was performed using GraphPad 9.0.

## Results

### Correlation between baseline characteristics and peripheral inflammatory factors

Baseline characteristics of 124 patients with advanced NSCLC who were treated with ICIs as first-line therapy are shown in Table 1. The majority of patients (89.5%) were male, and the median age was 65.0 years (43–81). 110 patients (88.7%) had an ECOG PS score of 0–1, and 14 patients (11.3%) had a score of ≥ 2. 67 patients (54.0%) had a smoking history. 51(41.1%) adenocarcinoma patients and 67(54.0%) squamous patients were involved. A total of 23 patients (18.5%) demonstrated PD-L1 expression ≥ 50%, 18 patients (14.5%) exhibited PD-L1 expression ranging from 1 to 49%, 8 patients (6.5%) showed PD-L1 expression < 1%, and 75 patients (60.5%) had an unknown PD-L1 expression status. There were 7 patients (5.6%) with brain metastasis and 18 patients (14.5%) with liver metastasis. In the first-line treatment, 112 patients (90.3%) received ICIs in combination with chemotherapy. 111 patients (89.5%) were treated with PD-1 inhibitors, and 13 patients (10.5%) were treated with PD-L1 inhibitors.

**Table 1:** Association between baseline characteristics and peripheral inflammatory factors.

Characteristic	N (%)	NLR			LMR			SII		
		< 3.61	≥ 3.61	*P* value	< 1.34	≥ 1.34	*P* value	< 1722.60	≥ 1722.60	*P* value
Age										
< 65	59(47.6%)	38 (64.4%)	21 (35.6%)	0.041*	4 (6.8%)	55 (93.2%)	0.053	50 (84.7%)	9 (15.3%)	0.094
≥ 65	65 (52.4%)	30 (46.2%)	35 (53.8%)		12 (18.5%)	53 (81.5%)		47 (72.3%)	18 (27.7%)	
Gender										
Male	111 (89.5%)	58 (52.3%)	53 (47.7%)	0.091	16 (14.4%)	95 (85.6%)	0.215	86 (77.5%)	25 (22.5%)	0.732
Female	13 (10.5%)	10 (76.9%)	3 (23.1%)		0 (0.0%)	13 (100.0%)		11 (84.6%)	2 (15.4%)	
ECOG										
0–1	110 (88.7%)	67 (60.9%)	43 (39.1%)	0.000*	13 (11.8%)	97 (88.2%)	0.389	87 (79.1%)	23 (20.9%)	0.503
≥ 2	14 (11.3%)	1 (7.1%)	13 (92.9%)		3 (21.4%)	11 (78.6%)		10 (71.4%)	4 (28.6%)	
Smoking history										
Yes	67 (54.0%)	32 (47.8%)	35 (52.2%)	0.086	9 (13.4%)	58 (86.6%)	0.849	46 (68.7%)	21 (31.3%)	0.005*
No	57 (46.0%)	36 (63.2%)	21 (36.8%)		7 (12.3%)	50 (87.7%)		51 (89.5%)	6 (10.5%)	
Histology										
Adenocarcinoma	51 (41.1%)	30 (58.8%)	21 (41.2%)	0.228	6 (11.8%)	45 (88.2%)	0.551	41 (80.4%)	10 (19.6%)	0.866
Squamous	67 (54.0%)	33 (49.3%)	34 (50.7%)		10 (14.9%)	57 (85.1%)		51 (76.1%)	16 (23.9%)	
Others	6 (4.8%)	5 (83.3%)	1 (16.7%)		0 (0.0%)	6 (100.0%)		5 (83.3%)	1 (16.7%)	
PD-L1 expression										
≥ 50%	23 (18.5%)	9 (39.1%)	14 (60.9%)	0.248	4 (17.4%)	19 (82.6%)	0.793	16 (69.6%)	7 (30.4%)	0.436
1–49%	18 (14.5%)	9 (50.0%)	9 (50.0%)		2 (11.1%)	16 (88.9%)		13 (72.2%)	5 (27.8%)	
< 1%	8 (6.5%)	6 (75.0%)	2 (25.0%)		0 (0.0%)	8 (100.0%)		6 (75.0%)	2 (25.0%)	
Unknown	75 (60.5%)	44 (58.7%)	31 (41.3%)		10 (13.3%)	65 (86.7%)		62 (82.7%)	13 (17.3%)	
Brain metastasis										
No	117 (94.4%)	64 (54.7%)	53 (45.3%)	1.000	15 (12.8%)	102 (87.2%)	1.000	91 (77.8%)	26 (22.2%)	1.000
Yes	7 (5.6%)	4 (57.1%)	3 (42.9%)		1 (14.3%)	6 (85.7%)		6 (85.7%)	1 (14.3%)	
Liver metastasis										
No	106 (85.5%)	62 (58.5%)	44 (41.5%)	0.047*	12 (11.3%)	94 (88.7%)	0.249	85 (80.2%)	21 (19.8%)	0.221
Yes	18 (14.5%)	6 (33.3%)	12 (66.7%)		4 (22.2%)	14 (77.8%)		12 (66.7%)	6 (33.3%)	
Option of treatment										
ICIs	12 (9.7%)	4 (33.3%)	8 (66.7%)	0.115	6 (50.0%)	6 (50.0%)	0.000*	8 (66.7%)	4 (33.3%)	0.407
ICIs + chemotherapy	112 (90.3%)	64 (57.1%)	48 (42.9%)		10 (8.9%)	102 (91.1%)		89 (79.5%)	23 (20.5%)	
Types of ICIs										
PD-1 inhibitors	111 (89.5%)	59 (53.2%)	52 (46.8%)	0.270	14 (11.9%)	97 (87.4%)	0.675	86 (77.5%)	25 (22.5%)	0.732
PD-L1 inhibitors	13 (10.5%)	9 (69.2%)	4 (30.8%)		2 (15.4%)	11 (84.6%)		11 (84.6%)	2 (15.4%)	

CR, complete response; PR, partial mitigation; SD, stable disease; PD, disease progression; ECOG, Eastern Cooperative Oncology Group; ICIs, Immune checkpoint inhibitors; NLR, neutrophil to lymphocyte ratio; LMR, lymphocyte to monocyte ratio; SII, Systemic inflammatory index. *, *p* < 0.05.

Age (*p* = 0.041), ECOG PS score (*p* = 0.000), and liver metastasis (*p* = 0.047) were found to be associated with NLR. The option of treatment was found to be associated with LMR (*p* < 0.001). Statistically significant differences were found between SII and smoking (*p* = 0.005). There were no statistically significant differences between peripheral inflammatory factors and other baseline characteristics.

### Efficacy of first-line ICIs in advanced driver gene-negative NSCLC

**Fig. 2:**
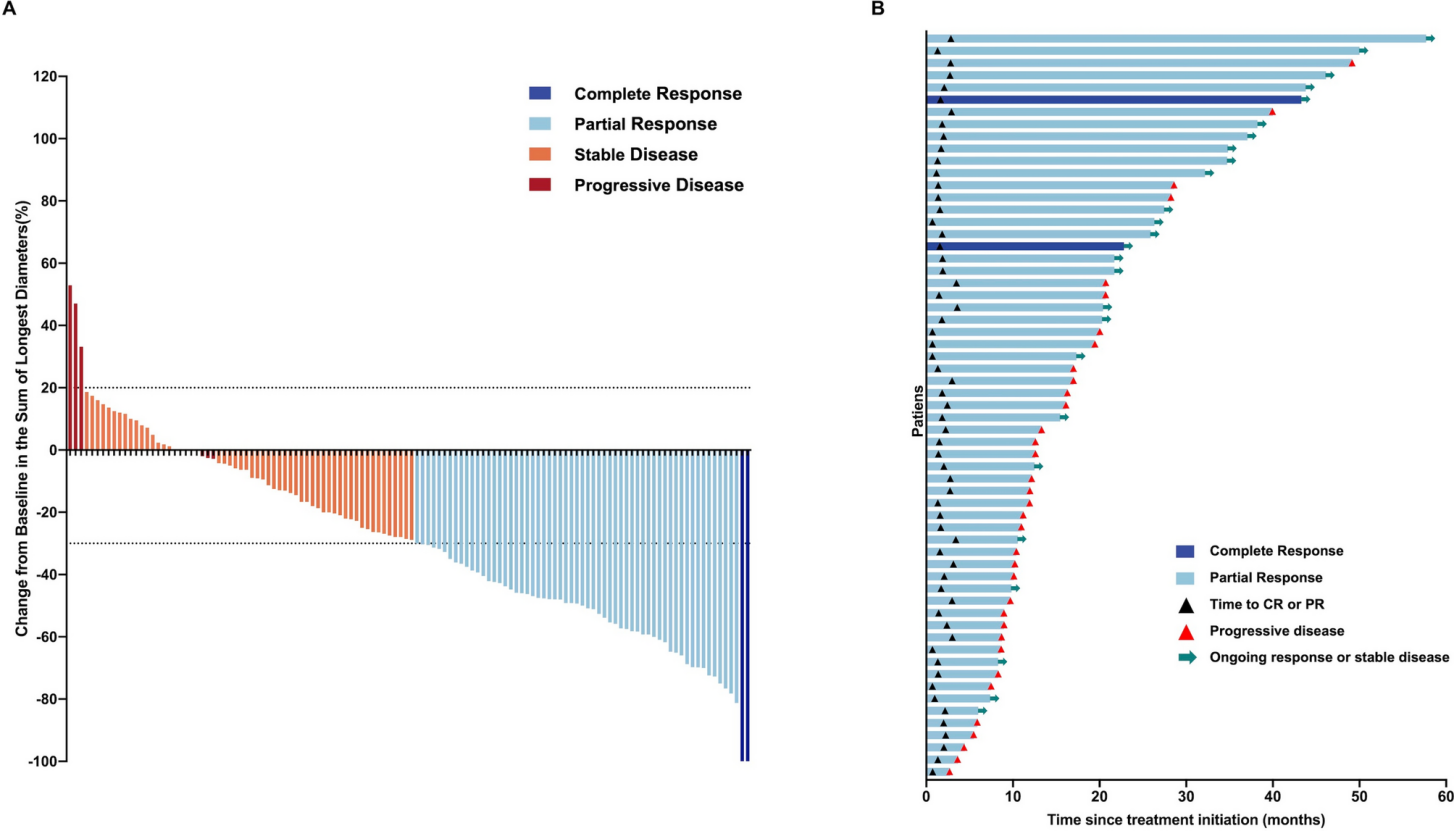
Response and duration of ICIs as first-line therapy in advanced driver gene-negative NSCLC. (**A**) Best change in the sum of the longest diameters of target lesions from baseline over the treatment period by RECIST response. (**B**) Time to and duration of response in patients with confirmed complete response or partial response (n = 61).

**Fig. 3:**
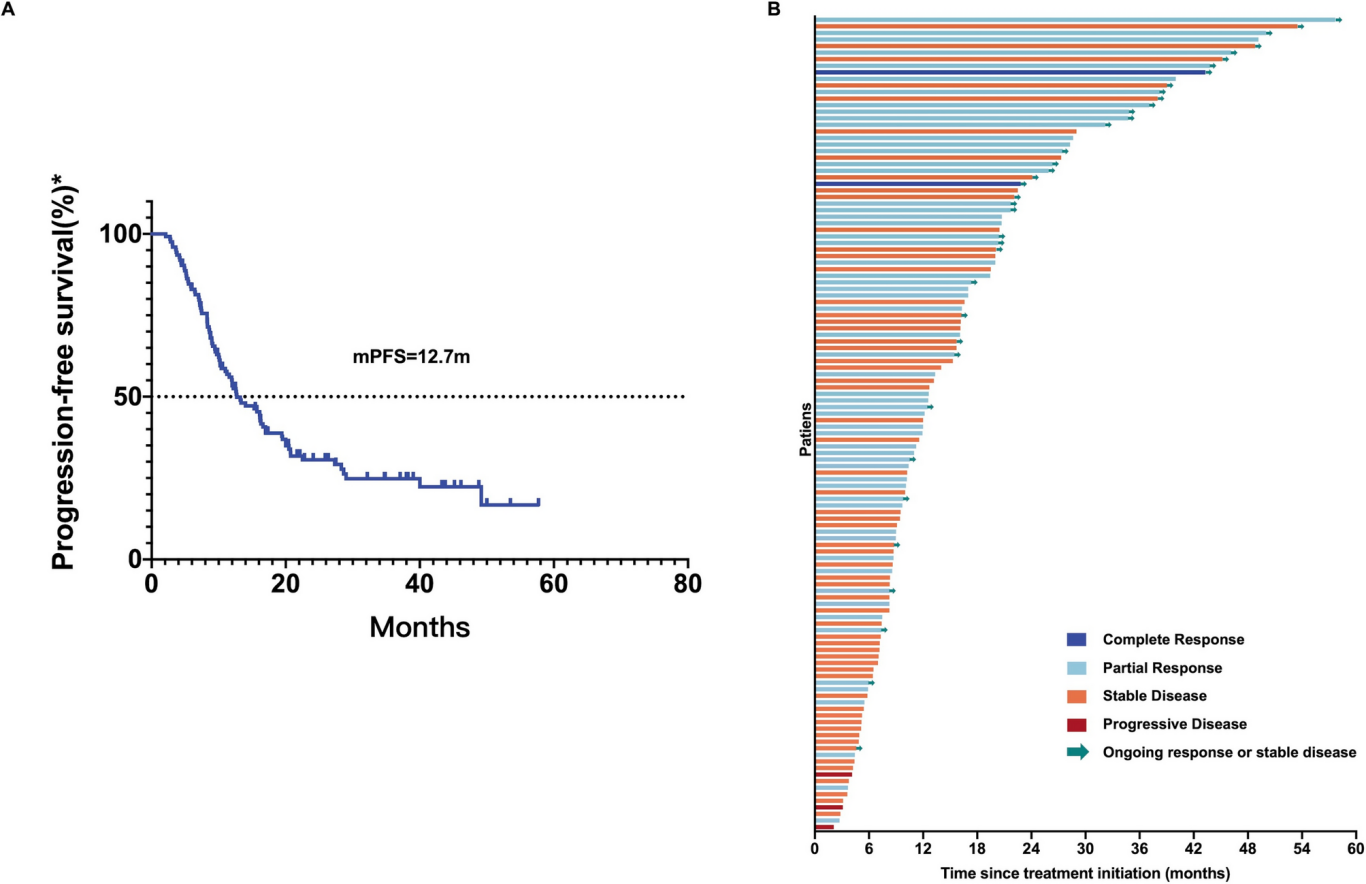
PFS for advanced driver gene-negative NSCLC treated with ICIs as first-line therapy. (**A**) Kaplan–Meier curves of PFS for advanced driver gene-negative NSCLC treated with ICIs as first-line therapy. (**B**) Duration of treatment in advanced driver gene-negative NSCLC treated with ICIs as first-line therapy.

The median observation period of this study was 34.7 (2.1–57.7) months. 2 patients (1.6%) achieved CR, 59 patients (47.6%) exhibited PR, 60 patients (48.4%) experienced SD, and 3 patients (2.4%) demonstrated PD (Table 2, Fig. 2A). The ORR at 12 weeks was 49.2%, and the DCR was 97.6%. Inter-group differences in the ORRs and DCRs were observed in Table 3. For the whole cohort included in the analysis, the median duration of response (DoR) was 13.7 months (range: 2.0–54.9, Fig. 2B). The median PFS was 12.7 months (95% CI 9.181–16.219, Fig. 3).

**Table 2:** Treatment response of ICIs as first-line therapy for advanced driver-negative NSCLC.

Response to ICIs	N (%)
CR	2 (1.6%)
PR	59 (47.6%)
SD	60 (48.4%)
PD	3 (2.4%)
ORR	49.2%
DCR	97.6%

CR, complete response; PR, partial mitigation; SD, stable disease; PD, disease progression; ORR, objective effective rate; DCR, disease control rate.

**Table 3:** Associations between peripheral inflammatory factors and response to ICIs.

	CR + PR	SD + PD	*P* value	CR + PR + SD	PD	*P* value
Age						
< 65	32 (54.2%)	27 (45.8%)	0.284	58 (98.3%)	1 (1.7%)	1.000
≥ 65	29 (44.6%)	36 (55.4%)		63 (96.9%)	2 (3.1%)	
Gender						
Male	56 (50.5%)	55 (49.5%)	0.413	108 (97.3%)	3 (2.7%)	1.000
Female	5 (38.5%)	8 (61.5%)		13 (100.0%)	0 (0.0%)	
ECOG						
0–1	53 (48.2%)	57 (51.8%)	0.528	107 (97.3%)	3 (2.7%)	1.000
≥ 2	8 (57.1%)	6 (42.9%)		14 (100.0%)	0 (0.0%)	
Smoking history						
Yes	38 (56.7%)	29 (43.3%)	0.069	67 (100.0%)	0 (0.0%)	0.094
No	23 (40.4%)	34 (59.6%)		54 (94.7%)	3 (5.3%)	
Histology						
Adenocarcinoma	23 (45.1%)	28 (54.9%)	0.764	49 (96.1%)	2 (3.9%)	0.030*
Squamous	35 (52.2%)	32 (47.8%)		67 (100.0%)	0 (0.0%)	
Others	3 (50.0%)	3 (50.0%)		5 (83.3%)	1 (16.7%)	
PD-L1 expression						
≥ 50%	13 (56.5%)	10 (43.5%)	0.647	23 (100.0%)	0 (0.0%)	0.313
1–49%	7 (38.9%)	11 (61.1%)		18 (100.0%)	0 (0.0%)	
< 1%	3 (37.5%)	5 (62.5%)		7 (87.5%)	1 (12.5%)	
Unknown	38 (50.7%)	37 (49.3%)		73 (97.3%)	2 (2.7%)	
Brain metastasis						
No	58 (49.6%)	59 (50.4%)	1.000	114 (97.4%)	3 (2.6%)	1.000
Yes	3 (42.9%)	4 (57.1%)		7 (100.0%)	0 (0.0%)	
Liver metastasis						
No	52 (49.1%)	54 (50.9%)	0.941	104 (98.1%)	2 (1.9%)	0.378
Yes	9 (50.0%)	9 (50.0%)		17 (94.4%)	1 (5.6%)	
Option of treatment						
ICIs	5 (41.7%)	7 (58.3%)	0.583	12 (100.0%)	0 (0.0%)	1.000
ICIs + chemotherapy	56 (50.0%)	56 (50.0%)		109 (97.3%)	3 (2.7%)	
Types of ICIs						
PD-1 inhibitors	53 (47.7%)	58 (52.3%)	0.347	109 (98.2%)	2 (1.8%)	0.285
PD-L1 inhibitors	8 (61.5%)	5 (38.5%)		12 (92.3%)	1 (7.7%)	
Baseline NLR						
NLR < 3.61	33 (48.5%)	35 (51.5%)	0.871	67 (98.5%)	1 (1.5%)	0.589
NLR ≥ 3.61	28 (50.0%)	28 (50.0%)		54 (96.4%)	2 (3.6%)	
NLR at 6 weeks						
NLR < 2.72	37 (52.1%)	34 (47.9%)	0.452	70 (98.6%)	1 (1.4%)	0.575
NLR ≥ 2.72	24 (45.3%)	29 (54.7%)		51 (96.2%)	2 (3.8%)	
NLR ratio						
NLR ratio < 0.68	34 (61.8%)	21 (38.2%)	0.012*	54 (98.2%)	1 (1.8%)	1.000
NLR ratio ≥ 0.68	27 (39.1%)	42 (60.9%)		67 (97.1%)	2 (2.9%)	
Baseline LMR						
LMR < 1.34	7 (43.8%)	9 (56.3%)	0.641	16 (100.0%)	0 (0.0%)	1.000
LMR ≥ 1.34	54 (50.0%)	54 (50.0%)		105 (97.2%)	3 (2.8%)	
LMR at 6 weeks						
LMR < 2.58	29 (48.3%)	31 (51.7%)	0.853	58 (96.7%)	2 (3.3%)	0.610
LMR ≥ 2.58	32 (50.0%)	32 (50.0%)		63 (98.4%)	1 (1.6%)	
LMR ratio						
LMR ratio < 1.38	44 (46.8%)	50 (53.2%)	0.347	92 (97.9%)	2 (2.1%)	0.568
LMR ratio ≥ 1.38	17 (56.7%)	13 (43.3%)		29 (96.7%)	1 (3.3%)	
Baseline SII						
SII < 1722.60	48 (49.5%)	49 (50.5%)	0.902	95 (97.9%)	2 (2.1%)	0.525
SII ≥ 1722.60	13 (48.1%)	14 (51.9%)		26 (96.3%)	1 (3.7%)	
SII at 6 weeks						
SII < 531.26	35 (61.4%)	22 (38.6%)	0.012*	56 (98.2%)	1 (1.8%)	1.000
SII ≥ 531.26	26 (38.8%)	41 (61.2%)		65 (97.0%)	2 (3.0%)	
SII ratio						
SII ratio < 0.74	42 (58.3%)	30 (41.7%)	0.017*	71 (98.6%)	1 (1.4%)	0.571
SII ratio ≥ 0.74	19 (36.5%)	33 (63.5%)		50 (96.2%)	2 (3.8%)	

CR, complete response; PR, partial mitigation; SD, stable disease; PD, disease progression; ECOG, Eastern Cooperative Oncology Group; ICIs, Immune checkpoint inhibitors; NLR, neutrophil to lymphocyte ratio; LMR, lymphocyte to monocyte ratio; SII, Systemic inflammatory index. *p < 0.05.

### Relationship between peripheral inflammatory factors and response to ICIs.

**Fig. 4:**
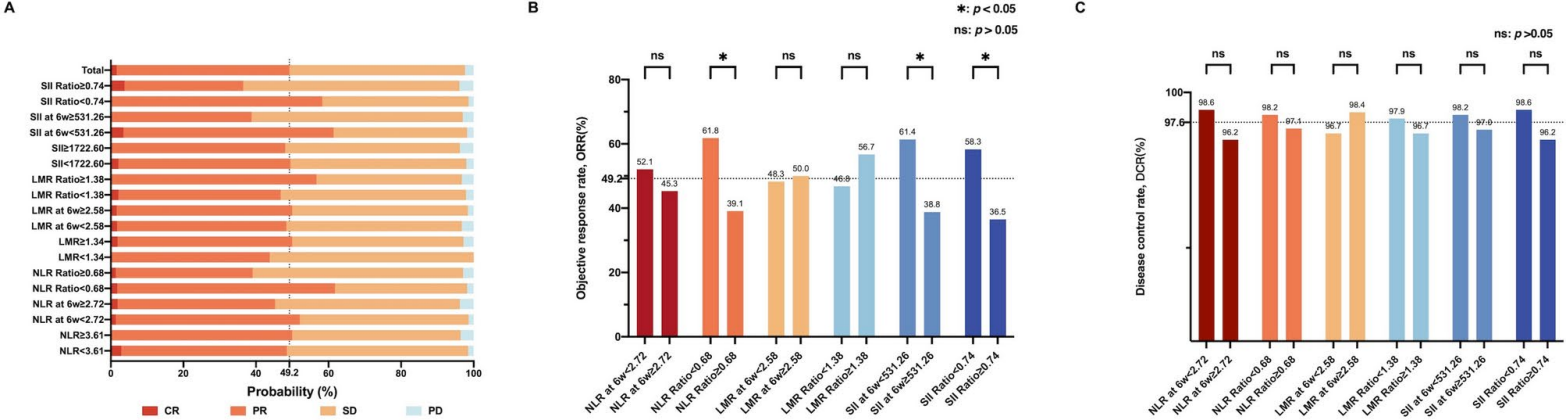
Response rates corresponding to NLR, LMR, and SII. (**A**) Treatment response related to NLR, LMR, and SII. (**B**) Statistical associations between NLR, LMR, SII, and objective response rate. (**C**) Statistical associations between NLR, LMR, SII, and disease control rate.

Patients with a baseline NLR ratio < 0.68 had the highest ORR of 61.8%, whereas patients with an SII ratio ≥ 0.74 had the lowest ORR of only 36.5%. NLR ratio, SII at 6 weeks, and SII ratio were significantly correlated with the ORR of ICIs therapy. Patients with an NLR ratio < 0.68 had a higher ORR than those with an NLR ratio ≥ 0.68 (61.8% vs. 39.1%; *p* = 0.012). Patients with an SII at 6 weeks < 531.26 had a higher ORR than those with an SII at 6 weeks $\ge$ 531.26 (61.4% vs. 38.8%; *p* = 0.012). Patients with an SII ratio < 0.74 had a higher ORR than those with an SII ratio ≥ 0.74 (58.3% vs. 36.5%; *p* = 0.017). These were shown in Fig. 4B. Response rates corresponding to NLR, LMR, and SII are shown in Fig. 4A. There were no statistically significant differences observed between DCR and NLR, LMR, or SII (*p* > 0.05) (Fig. 4C).

There were significant differences in the DCR associated with histology. Patients with squamous had a higher DCR than those with adenocarcinoma and others (100.0% vs. 96.1% vs. 83.3%; *p* = 0.030). There were no significant differences observed in ORR and other baseline characteristics. The PD-L1 expression was known in 49 of 124 patients. With respect to them, patients with PD-L1 ≥ 50% had the highest ORR of 56.5%, whereas patients with PD-L1 < 1% had the lowest ORR of 37.5% (*p* = 0.647).

### Relationship between peripheral inflammatory factors and PFS to ICIs

**Fig. 5:**
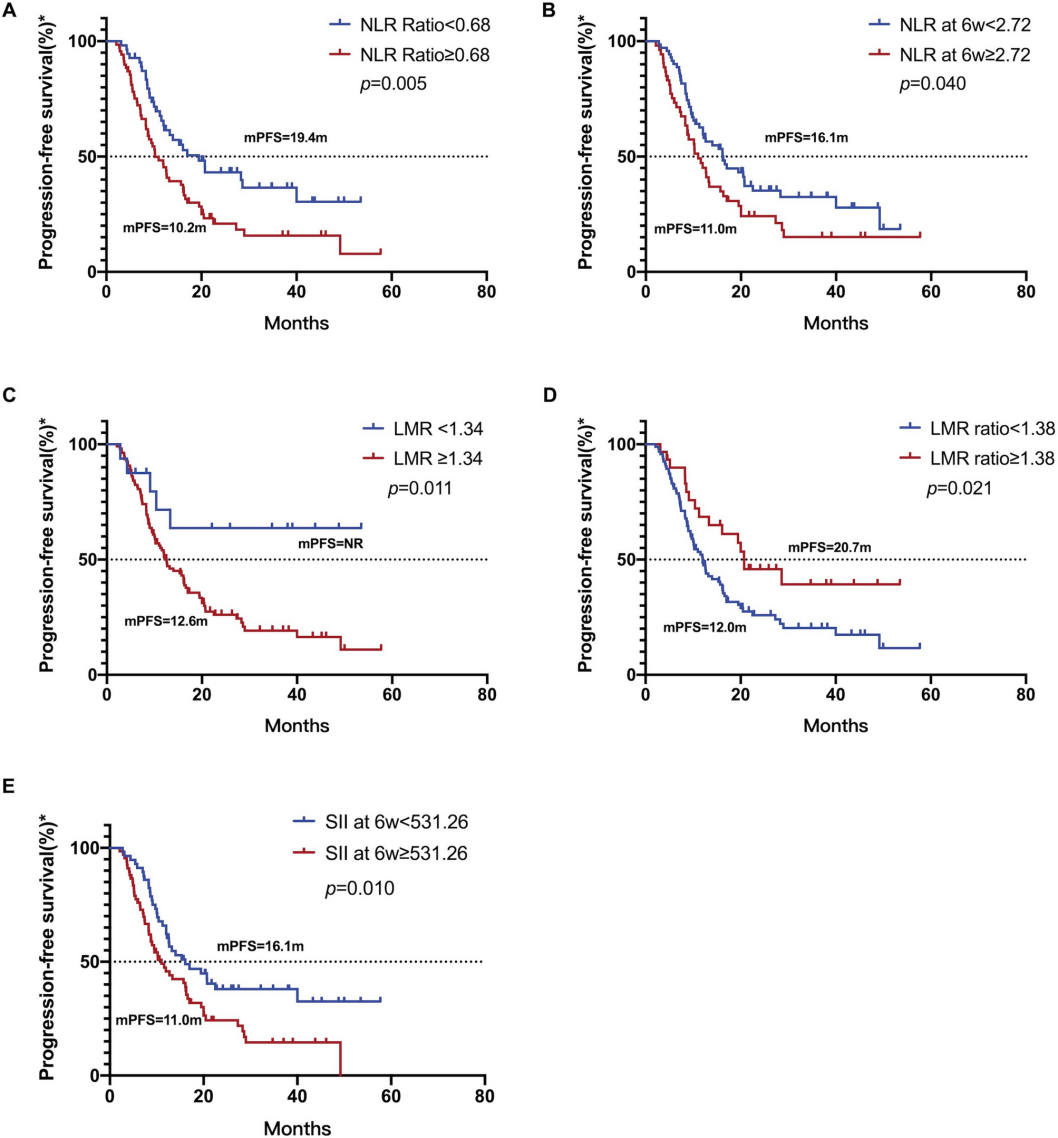
Kaplan–Meier curves of PFS for patients treated with ICIs as first-line therapy in relation to clinical parameters. (**A**) Kaplan–Meier curves of PFS for NSCLC patients treated with ICIs as first-line therapy in relation to NLR ratio. (**B**) Kaplan–Meier curves of PFS for NSCLC patients treated with ICIs as first-line therapy in relation to NLR at 6 weeks. (**C**) Kaplan–Meier curves of PFS for NSCLC patients treated with ICIs as first-line therapy in relation to baseline LMR. (**D**) Kaplan–Meier curves of PFS for NSCLC patients treated with ICIs as first-line therapy in relation to LMR ratio. (**E**) Kaplan–Meier curves of PFS for NSCLC patients treated with ICIs as first-line therapy in relation to SII at 6 weeks.

Univariate and multivariate analyses were conducted to identify variables associated with PFS in patients receiving ICIs (Table 4). Regarding peripheral inflammatory factors, NLR at 6 weeks, NLR ratio, baseline LMR, LMR ratio, and SII at 6 weeks were found to be associated with PFS. The results indicated that patients with an NLR ratio < 0.68 had significantly longer PFS compared to those with an NLR ratio ≥ 0.68 (*p* = 0.005, Fig. 5A). Similar results were observed for patients with an NLR at 6 weeks < 2.72 (*p* = 0.040, Fig. 5B), a baseline LMR < 1.34 (*p* = 0.011, Fig. 5C), an SII at 6 weeks < 531.26 (*p* = 0.010, Fig. 5E), all of whom had improved PFS. Additionally, patients with a LMR ratio ≥ 1.38 (*p* = 0.021, Fig. 5D) were significantly associated with prolonged PFS. Furthermore, univariate analysis showed that ECOG PS (*p* = 0.031) and smoking history (*p* = 0.008) were significantly correlated with PFS. Patients with ECOG PS of 0–1, and patients with a history of smoking were found to have longer PFS.

**Table 4:** Univariate and multivariate analyses of PFS with peripheral inflammatory factors.

Variable	Sample	Univariate analyses		Multivariate analyses	
		Median PFS (month)	*P* value	HR (95%CI)	*P* value
Patients	124	12.7			
Age					
< 65	59	11.9	0.291		
≥ 65	65	16.6			
Gender					
Male	111	14.0	0.057*		
Female	13	10.2			
ECOG					
0–1	110	14.0	0.031*		
≥ 2	14	8.7			
Smoking history					
Yes	57	16.3	0.008*	1.719 (1.121–2.637)	0.013**
No	67	10.1			
Histology					
Adenocarcinoma	51	14.0	0.932		
Squamous	67	12.0			
Others	6	9.0			
PD-L1 expression					
≥ 50%	23	20.7	0.082*		
1–49%	18	9.0			
< 1%	8	10.0			
Unknown	75	15.7			
Brain metastasis					
No	117	12.7	0.162*		
Yes	7	–			
Liver metastasis					
No	106	13.2	0.302		
Yes	18	11.0			
Option of treatment					
ICIs	12	22.5	0.126*		
ICIs + chemotherapy	112	12.6			
Types of ICIs					
PD-1 inhibitors	111	12.6	0.325		
PD-L1 inhibitors	13	20.0			
Baseline NLR					
NLR < 3.61	68	15.3	0.942		
NLR ≥ 3.61	56	11.6			
NLR at 6 weeks					
NLR < 2.72	71	16.1	0.040*		
NLR ≥ 2.72	53	11.0			
NLR ratio					
NLR ratio < 0.68	55	19.4	0.005*		
NLR ratio ≥ 0.68	69	10.2			
Baseline LMR					
LMR < 1.34	16	–	0.011*	3.102 (1.249–7.704)	0.015**
LMR ≥ 1.34	108	12.6			
LMR at 6 weeks					
LMR < 2.58	60	15.7	0.183*		
LMR ≥ 2.58	64	12.0			
LMR ratio					
LMR ratio < 1.38	94	12.0	0.021*		
LMR ratio ≥ 1.38	30	20.7			
Baseline SII					
SII < 1722.60	97	12.6	0.514		
SII ≥ 1722.60	27	14.0			
SII at 6 weeks					
SII < 531.26	57	16.1	0.010*	1.706 (1.103–2.638)	0.016**
SII ≥ 531.26	67	11.0			
SII ratio					
SII ratio < 0.74	72	14.0	0.109*		
SII ratio ≥ 0.74	52	12.0			

*Included in multivariate analysis.

**p < 0.05 in multivariate analysis.

ECOG, Eastern Cooperative Oncology Group Performance Status; ICIs, Immune checkpoint inhibitors; NLR, neutrophil to lymphocyte ratio; LMR, lymphocyte to monocyte ratio; SII, Systemic inflammatory index; PFS, progression-free survival; HR, hazard ratio; CI, confidence interval.

Peripheral inflammatory factors and baseline characteristics with a *p* value < 0.20 in the univariate analysis were included in the multivariate analysis. The results indicated that baseline LMR (HR = 3.102, 95%CI 1.249–7.704, *p* = 0.015), SII at 6 weeks (HR = 1.706, 95% CI 1.103–2.638, *p* = 0.016), and smoking history (HR = 1.719, 95% CI 1.121–2.637, *p* = 0.013) were identified as independent prognostic factors for PFS (Table 4). Patients with a baseline LMR < 1.34, SII at 6 weeks < 531.26, and those with a history of smoking were independently associated with improved PFS.

## Discussion

ICIs have greatly changed treatment paradigms and have been pivotal in the treatment of advanced NSCLC patients^23^. The PD-L1 expression level, determined by immunohistochemistry, was the first clinically validated predictive biomarker that has been translated into clinical practice^24,25^. It is generally believed that high PD-L1 expression is related to an increased response rate and clinical benefit in ICIs^26–28^. However, a high number of patients still do not respond to ICIs, and resistance development is inevitable in most cases. In addition, a proportion of patients with negative PD-L1 expression can also benefit from ICIs^29^. In the real world, a large proportion of patients are unable to detect the expression of PD-L1 because they do not have enough tissue or for other reasons. Thus, there is an urgent need for better biomarkers that could effectively predict the efficacy and prognosis of ICIs in advanced NSCLC. In this study, we retrospectively studied the correlation between peripheral inflammatory factors, efficacy and prognosis in first-line ICIs for advanced driver gene-negative NSCLC. Among them, patients with low NLR, high LMR ratio, or low SII had better efficacy and prognosis when they received first-line ICIs. ORRs were higher in patients with an NLR ratio < 0.68, SII at 6 weeks < 531.26 and SII ratio < 0.74. DCRs were significantly higher in squamous groups. This study has also shown that baseline LMR < 1.34 and SII at 6 weeks < 531.26 are independent factors significantly associated with PFS in this cohort. These associations suggest that NLR, LMR, and SII may be predictive biomarkers for NSCLC patients treated with ICIs.

Inflammation predisposes to the development of cancer and promotes all stages of tumorigenesis^30^. Inflammation is an important component of the tumor microenvironment (TME), and changes in inflammatory cells may affect the occurrence and development of tumor. High levels of tumor-associated neutrophils (TANs) and tumor-associated macrophages (TAMs) have been described as mediators in tumor progression because they promote the growth of tumor cells by inhibiting apoptosis and assisting angiogenesis, which promotes the formation of metastases^31,32^. Lymphocytes are involved in the regulation of host cell-mediated immunity, which is essential for destroying residual tumor cells and related micrometastases^33^. Monocytes may be reprogrammed to differentiate into TAMs and mononuclear dendritic cells, which leads to tumor progression^34^. Platelets are able to promote tumor growth and metastasis^35^. Thus, peripheral inflammation factors may reflect cancer-related inflammation and antitumor immune responses. NLR, LMR, and SII have been shown to be associated with the prognosis of a variety of cancers^36–38^. Some retrospective studies of NSCLC showed that the increased NLR was consistently related to an inferior PFS and OS^39,40^. However, the use of retrospective data and different cut-offs for NLR have limited its validation as a biomarker. Several studies have been published focusing on the relationship between NLR and the efficacy and prognosis of ICIs, but there has been less research on the efficacy and prognosis of LMR and SII in advanced NSCLC^41–43^.

This study demonstrated that the efficacy and prognosis of patients with an NLR at 6 weeks < 2.72 and an NLR ratio < 0.68 were improved. For LMR, patients with an LMR ratio ≥ 1.38 had a higher ORR and a longer PFS. The results for SII showed that elevated values were associated with worse treatment response and poor survival. Furthermore, our study indicated that LMR and SII were independent factors in PFS, with only SII remaining significant in ORR, univariate analyses, and multivariate analyses. The interaction between circulating tumor cells and platelets can support circulating tumor cells by protecting them from immune detection, which further protects cancer cells^44^. Therefore, the addition of the platelet count to the calculation of the SII may explain why it appears to be a better indicator of prognosis. Wen S et al. demonstrated that decreased NLR at 6 weeks, decreased derived NLR (dNLR) at 6 weeks, and increased LMR at 12 weeks were associated with increased ORR in NSCLC patients after PD-1 inhibitor-based immunotherapy^45^. It has been reported that NLR at 6 weeks < 5 and SII at 6 weeks < 730 were significantly associated with better ORR, longer PFS, and longer OS in patients with advanced NSCLC treated with anti-PD-1 antibodies^46^. In a retrospective analysis of 81 NSCLC patients who received atezolizumab monotherapy, the pretreatment inflammatory factors, specifically high NLR, low LMR, and high PLR, were significantly associated with shorter PFS and OS^47^. Our findings are consistent with previous reports of NLR and LMR in patients with NSCLC treated with ICIs, and suggest that SII appears superior to NLR and LMR as a comprehensive biomarker in patients with advanced NSCLC treated with first-line ICIs. In addition, since smoking is associated with an increase in TMB, it is possible that smokers could obtain more benefits from ICIs^48^. This may explain the correlation between a worse prognosis and a non-smoking history.

The main mechanism of ICIs is to change TME or immune cells, so that the immune system can achieve the purpose of killing tumors^49^. In the TME, tumor cells reprogram surrounding stromal cells, leading to an inflammatory response with the expansion and recruitment of different immune cells to support cancer progression^50^. ICIs cause the activation of CD8+ T cells, reduce monocyte reprogramming, and increase lymphocyte numbers. This increases monocyte recruitment and infiltration in TME and also increases anti-tumor CD8+ T cell numbers in peripheral blood cells^51^. Thus, the complex and dynamic interaction between cancer and the immune system during ICI treatments can hardly be predicted by the evaluation of a single biomarker.

Peripheral inflammatory factors may reflect a broad interaction between systemic inflammation and overall immune function, and in the context of cancer, they may serve as a proxy for the equilibrium between tumor-mediated inflammation and antitumor immunity. However, most prior studies have focused on baseline peripheral inflammatory factors without clear evidence that these reflect distinct immune surveillance states in the TME^52–54^. Yuan et al. found a high correlation between the LMR/NLR and tumor immune microenvironment in gastric cancer. The density of exhausted CD8 T cells was negatively associated with LMR and positively associated with NLR. NLR and LMR were significantly associated with PFS and OS. Additionally, M1 macrophages were suggested to be associated with PLR and SII, which may help distinguish immune tolerance and resistance^55^. Hwang et al. demonstrated that NLR dynamics may be an indirect predictive marker of an emerging antitumor immune response during immunotherapy and favorable clinical outcomes^56^. Zheng et al. showed that a decrease in MLR was associated with improved treatment outcomes in patients with stage IIIB-IV NSCLC treated with PD-1 inhibitors combined with chemotherapy as first-line therapy^43^. Here we have shown that decreased NLR and SII at 6 weeks were associated with higher ORR and longer PFS. Taken together, this suggests that post-treatment peripheral inflammatory factors are more indicative of the changes in the TME and anti-tumor immune response in patients compared to the baseline peripheral inflammatory factors. While our results support the prognostic significance of peripheral inflammatory factors, they cannot be used as predictors for the discontinuation of treatment in clinical practice, and the termination of treatment still needs to be comprehensively judged in combination with clinical practice. Further studies are needed to confirm the predictive value of changes in these inflammatory markers for shorter-term efficacy and longer-term efficacy in advanced NSCLC patients treated with ICIs.

So far, the results of phase III clinical studies of first-line ICIs for advanced NSCLC show that the ORR of ICIs is between 26 and 75%, and the median PFS is between 4.2 and 11.3 months^5^. In this study, the ORR was 49.2%, and the DCR was 97.6%. The median PFS was 12.7 months, and the DoR was 13.7 months. The combined results of previous studies and this study suggest that ICIs therapy is an option for patients with advanced driver gene-negative NSCLC. The ORR, DCR, and PFS of patients in the ICIs monotherapy group were all better than those in previous studies, which may be related to the high PD-L1 expression in the included patients who received monotherapy, the small number of included cases, and the short follow-up time.

In our study, PD-L1 expression was unknown in the majority of patients, and peripheral inflammatory factors showed potential as biomarkers of efficacy and prognosis. At the same time, the detection of peripheral inflammatory factors has the advantages of easy access, lower cost, noninvasive nature, and repeatability during treatment. Peripheral inflammatory factors may serve as a complement to PD-L1 expression, providing the possibility of individualized and precise treatment for patients with driver gene-negative NSCLC. However, this retrospective study has several limitations. First of all, the small sample size of patients might not be sufficient to support the stability of the results. Second, due to the short follow-up time, most patients did not reach the survival endpoint event, which could not be statistically analyzed for overall survival. Then, peripheral inflammatory factors as biomarkers of ICIs still need to be further explored because factors such as inflammation, basal diseases, the occurrence of bone marrow suppression when combined with chemotherapy, and medications may have an impact on them. After that, the large number of patients included in ICIs plus chemotherapy limited the generalizability of the results, and large prospective studies in the future are necessary. Moreover, since PD-L1 expression was known in so few patients in our study, there remains ambiguity regarding the correlation between peripheral inflammatory factors and PD-L1 expression. Finally, further exploration of the correlation between TME and neutrophils, lymphocytes, and platelets is necessary to provide a theoretical basis for efficient and accurate differentiation of the benefit populations of ICIs in real-world clinical applications.

## Data Availability

The raw data supporting the conclusions of this article will be made available by the authors, without undue reservation. The data underlying this article will be shared on reasonable request to the corresponding author.
